# Olive oil from the 79 A.D. Vesuvius eruption stored at the Naples National Archaeological Museum (Italy)

**DOI:** 10.1038/s41538-020-00077-w

**Published:** 2020-11-02

**Authors:** Raffaele Sacchi, Adele Cutignano, Gianluca Picariello, Antonello Paduano, Alessandro Genovese, Francesco Siano, Genoveffa Nuzzo, Simonetta Caira, Carmine Lubritto, Paola Ricci, Alessia D’Auria, Gaetano Di Pasquale, Andrea Motta, Francesco Addeo

**Affiliations:** 1 Department of Agricultural Sciences, Unit of Food Science & Technology, University of Naples “Federico II”, Via Università 100, 80055 Portici (Naples), Italy; 2 Institute of Biomolecular Chemistry, National Research Council, ICB-CNR, Via Campi Flegrei 34, 80078 Pozzuoli (Naples), Italy; 3 Institute of Food Sciences, National Research Council, ISA-CNR, Via Roma 64, 83100 Avellino, Italy; 4 Department of Agricultural and Environmental Science, University of Bari “Aldo Moro”, Via Orabona 4, 70125 Bari, Italy; 5 Institute for the Animal Production System in the Mediterranean Environment, National Research Council, ISPAAM-CNR, Via Argine 1085, 80147 Naples, Italy; 6 Department of Environmental, Biological and Pharmaceutical Science and Technology, University of Campania “Luigi Vanvitelli”, Via Vivaldi 43, 81100 Caserta, Italy

**Keywords:** Fatty acids, Mass spectrometry

## Abstract

Using a range of chromatographic, spectroscopic, and mass spectrometric analytical techniques, we characterized one of the “edible items” found at the Vesuvius archeological sites and guarded at the National Archaeological Museum of Naples (MANN) in Naples, Italy. We authenticated the specimen contained in a glass bottle (Mann-S1 sample) as originally olive oil and mapped the deep evolution throughout its 2000 years of storage. Triacylglycerols were completely hydrolyzed, while the resulting (hydroxy) fatty acids had partly condensed into rarely found estolides. A complex pattern of volatile compounds arose mainly from breakdown of oleic acid. With excellent approximation, radiocarbon dating placed the find at the time of the Plinian Mount Vesuvius eruption in 79 A.D., indicating that Mann-S1 is probably the oldest residue of olive oil in the world found in bulk amount (nearly 0.7 L).

## Introduction

Pompeii and Herculaneum represent extraordinary windows into the life of the ancient Romans. The two prosperous towns were instantly buried by a thick layer of superheated pyroclastic material that erupted from Mount Vesuvius in 79 A.D., crystalizing into a concrete snapshot and long enshrined a moment in their everyday lives.

**Fig. 1:**
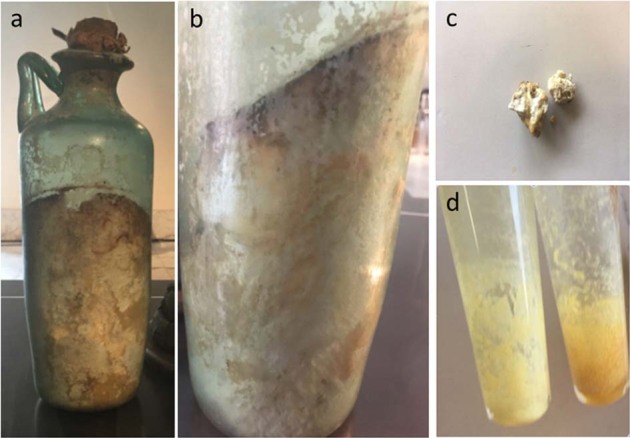
Item no. 313337 stored at the National Archaeological Museum of Naples (MANN). **a** Glass bottle containing Mann-S1; **b** detail of the organic content with inclined surface indicating an original liquid status; **c** sample used for chemical analyses (Mann-S1); **d** lipid extracts from Mann-S1.

Systematic excavations of the sites began with Prince d’Elboeuf in 1738 and continued with King Carlo of Bourbon, who dedicated several rooms of the Royal Palace of Portici (later the Herculaneum Museum) to the exhibition of archeologically interesting items (*Gabinetto de’ Preziosi*). Between 1805 and 1828, the collection was moved to the actual deposits of the Naples National Archaeological Museum (MANN, Naples, Italy)^1^. The MANN stores wealthy collections coming from the Vesuvius archeological excavations. The “edible items” (*Collezione dei Commestibili*) are remnants of food plants recently exposed in the *Res Rustica*, which is the first complete exhibition of the organic finds from Pompeii and Herculaneum. They include a large variety of charred seeds and fruits along with one loaf of bread. The collection also features kitchen tools for various domestic purposes and differently shaped glass bottles, such as the bottle used for this study. Most likely, 15 out of the 22 cataloged glass bottles coming from Vesuvius archeological sites contain solidified olive oil^2^. However, the identity and authenticity of the archeological organic matter must be validated. In fact, a recent radiocarbon datation of grape pomace (*Vitis vinifera*), recovered in the storerooms of the MANN, placed this material in the modern period, probably to the XVIII century, at the time of the first excavations (D’Auria et al., submitted), thereby suggesting the need for caution in authenticating the finds. When the MANN provided us with a sample of organic matter guarded in an almost-full glass bottle photographed in Fig. 1a, we imagined that the historian Pliny the Younger, who attended and described the Vesuvius eruption (Plinius Juvenis, Epistulae ad Familiares, 6, 16), had come to our food science laboratory (from which one can reach the ancient walls of Herculaneum by taking a 5-min walk), wondering if it was actually a “Campania Felix” olive oil of his time and, in this case, what had remained of that antique oil.

During the Roman Imperial age, olive oil was the main dietary vegetable oil; it was also used for lighting, therapy, pregnancy, and delivery (Soranus of Ephesus, Gynecology, Owsei Tempkin translator, Johns Hopkins University Press, 1956) as well as cosmetics (Pliny the Elder, *Naturalis Historia*, XVIII), such as a characteristic rose-scented olive oil^3^. Olive oil was produced at significant amounts in the “Campania Felix” territory and high-quality oils are described in Venafro, not far from the Vesuvius region (Marziale, Epigrammaton, XII, 63,101; Pliny, *Naturalis Historia*, XVIII, 111). However, the discovery of amphorae of the Northern African and Iberian kinds during excavations testifies to the fact that some of the olive oil was imported from *coloniae*.

Nicola Covelli first attempted the characterization of the organic contents of a glass bottles from Vesusius excavations in 1826, based on appearance and sensorial traits. One of the Covelli’s reports^2^ about presumed olive oil is transcribed in Supplementary Material.

More recent analyses with spectroscopic and chromatographic methods have established the lipid nature of organic traces in some archeological glass bottles, though the results appear inconclusive, leaving the residue chemically and chronologically yet uncharacterized^4–6^. Tanasi et al.^7^ analyzed residues of prehistoric lipids from Sicily by gas chromatography (GC)/mass spectrometry (MS) and nuclear magnetic resonance (NMR). However, the profound chemical modifications occurring throughout such a long time interval complicate the assessment of the nature of oils or fats, primarily because the fatty acid (FA) profile drastically changes due to the very different oxidation rate depending on the degree of unsaturation. Thus, GC/MS of FAs and NMR separately may not unambiguously discriminate the plant or animal origin of archeological fats^8,9^. In general, investigations have been prejudiced by the assumption that lipids can boast of exceptional time stability, disregarding the dramatic chemical modifications triggered by the initial severe thermal shock and the very long period of storage^4^.

In this work, for the first time to our knowledge, the authenticity and identity of an olive oil sample, hereinafter referred to as Mann-S1, which has been stored seemingly in its original glass bottle since 79 A.D., has been assessed through radiocarbon dating and detection of analytical biomarkers. The availability of a conspicuous amount of such a well-preserved specimen of organic material is rare and gave us the chance to map the molecular evolution that oil has undergone for almost 2000 years.

## Results

### Chemical composition of the archeological organic find

The assessment of the authenticity, composition, and identity of the Mann-S1 sample contained in the glass bottle under investigation (Fig. 1) required an integrated analytical strategy based on ultrahigh performance liquid chromatography–tandem mass spectrometry (UHPLC-MS/MS), matrix-assisted laser desorption ionization–time of flight (MALDI-TOF)/MS, NMR spectroscopy, high-resolution (HR)-GC of fatty acid methyl esters (FAMEs), determination of free FAs and peroxide value, attenuated total reflectance–Fourier transform infrared (ATR-FTIR), GC of sterols, solid phase microextraction (SPME)-GC/MS of volatile compounds and radiocarbon dating.

### Ultrahigh performance liquid chromatography–tandem mass spectrometry

**Fig. 2:**
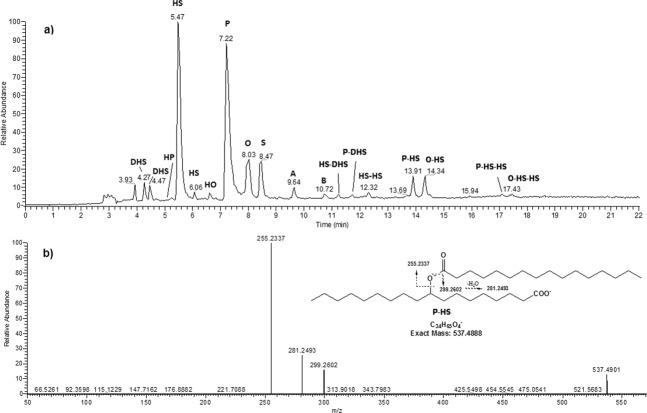
UHPLC-HR-ESI MS analysis of Mann-S1. **a** Representative total ion current negative ion mode chromatogram of lipids and **b** MS/MS-based assignment of the estolide P-HS on isolated ion at *m*/*z* 537.4901. The insert shows fragment ion identification of a putative P-HS isomer (9-hexadecanoyloxyoctadecanoic acid, PubChem CID: 72189985), although several other isomers are possible. Fatty acid acronyms: A arachidic acid (C20:0), B behenic acid (C22:0), O oleic acid (C18:1), P palmitic acid (C16:0), S stearic acid (C18:0), HO hydroxyoleic acid, HP hydroxypalmitic acid, HS hydroxystearic acid, DHS dihydroxystearic acid isomer.

Modern lipidomic approaches based on soft ionization MS techniques (i.e., ESI and MALDI) enable the direct identification of intact lipid components. The UHPLC-ESI-MS profile of the lipid material was performed in both positive and negative modes (Fig. 2a and Supporting Information). However, because sodium adducts [M + Na]^+^ ionized with lower efficiency in comparison with negative [M-H]^−^ ions, further investigations were carried out in negative polarity. The LC-ESI-MS^−^ profile was dominated by FA ions of palmitic (P) and monohydroxy stearic acid (HS), and to a minor extent stearic (S) and oleic (O) acids. Dihydroxystearic isomers and monohydroxyoleic (HO) acids occurred at lower levels, along with trace amounts of hydroxypalmitic (HP), eicosanoic (arachidic (A)), and docosanoic (behenic (B)) acids (Fig. 2a). In addition to FAs and their oxygenated products, a few minor peaks were detected in the UHPLC-MS trace. Surprisingly, in-depth interpretation of MS/MS data allowed for the disclosure of a family of branched oligomeric FA esters of hydroxyl FAs, also called estolides. Mann-S1 contained estolides exhibiting different levels of FA oxidation, such as dimeric hydroxystearic acids (HS-HS) at *m*/*z* 581.5168, hydroxystearic and palmitic acids (P-HS) at *m*/*z* 537.4910, and hydroxystearic and oleic acids (O-HS) at *m/z* 563.5067. These ions typically fragment at the level of the ester function, generating detectable product ions from both sides of the cleavage site (Fig. 2b). Table 1 reports a list of all annotated components with their respective fragments, including trimers consisting of two units of hydroxystearic acids with one unit of palmitic acid (P-HS-HS) at *m*/*z* 819.7473 or oleic acid (O-HS-HS) at *m*/*z* 845.7625, both detectable at higher retention times. Notably, no trace of glycerol-based lipids was detected.

**Table 1:** Fatty acids and their derivatives detected by UHPLC-ESIMS/MS in negative ion mode on sample Mann-S1.

Assignment	*t*_R_	Relative area (%)	Obs *m*/*z* [M-H]^−^	Calc *m*/*z* [M-H]^−^	MS^2^
DHS	4.27/4.47	3.3	315.2548	315.2541	297.24/279.23
HP	5.09	0.17	285.2442	285.2435	
HS	5.47/6.06	35.28	299.2600	299.2592	281.25
HO	6.65	0.71	297.2442	297.2435	279.23
P	7.22	38.01	255.2335	255.2330	
S	8.03	9.02	283.2651	283.2643	
O	8.47	6.41	281.2494	281.2486	
A	9.64	1.33	311.2965	311.2956	
B	10.72	0.42	339.3276	339.3269	
HS-DHS estolide	10.79/11.17	0.08	597.5116	597.5094	315.25/299.26/281.25
P-DHS estolide	11.51/11.66	0.12	553.4852	553.4832	315.25/297.24/255.23
HS-HS estolide	12.32	0.39	581.5168	581.5151	299.26/281.25
P-HS estolide	13.91	1.97	537.4901	537.4888	299.26/281.25/255.23
O-HS estolide	14.34	2.19	563.5067	563.5045	299.26/281.25
P-HS-HS estolide	17.11	0.3	819.7473	819.7447	563.50/537.49/299.26/281.25/255.23
O-HS-HS estolide	17.47	0.29	845.7625	845.7604	581.51/563.51/299.26/281.25

*DHS* dihydroxystearic acid isomers, *HS* hydroxystearic acid isomers, *HO* hydroxyoleic acid, *P* palmitic acid, *S* stearic acid, *O* oleic acid, *A* arachidic acid, *B* behenic acid.

Estolides are quite rare in nature. They have been reported as neo-formed compounds in several seed oils and, recently, in human adipose tissue and tears^10,11^. These compounds have attracted attention due to their physicochemical properties for a variety of potential applications, such as greases, plastics, inks, cosmetics, and viscosity controllers in food and lubricants; therefore, they are commonly produced by synthesis at high temperatures (205–210 °C) or strong acidic catalysis^12^. Several studies have described the formation of estolides from long-chain hydroxy-FAs, using immobilized commercial lipases or, more intriguingly, a submerged culture of *Pseudomonas* sp. grown on oleic acid^13,14^. Further analyses are needed to establish whether the formation of estolides in Mann-S1 was triggered by the temperature increase caused by the Vesuvius eruption or catalyzed enzymatically due to microbial contamination. An additional possibility lies in the slow chemical transformation of a naturally acid crude olive oil that occurred over a very long time scale.

### Matrix-assisted laser desorption ionization–time of flight MS

Due to matrix interference, MALDI MS is infrequently used for the analysis of relatively small molecules. Nevertheless, under appropriate experimental conditions, MALDI-TOF MS enables robust and reliable analysis of unfractionated FAs and their derivatives, consistent with the GC determination, as well as the simultaneous detection of mono-, di-, and tri-acylglycerols^15,16^. The MALDI-TOF MS spectrum of Mann-S1 is shown in Supplementary Material (Fig. S2). Using Na^+^ as a dopant, in the MALDI positive ion mode, FAs are detected as sodiated sodium carboxylates [RCOONa + Na]^+^ and can be identified matching the measured with expected molecular weights. In agreement with UHPLC-MS/MS data, the most intense signals were S (*m*/*z* 329.4), HO (*m*/*z* 343.4), and HS (*m*/*z* 345.4) FAs. Free palmitate (*m*/*z* 301.3) occurred as a minor component. Putative HP (*m*/*z* 317.3) as well as dihydroxy derivatives of oleic (*m*/*z* 359.3) and stearic (*m*/*z* 361.3) acids were also clearly detected. The intense signal at *m*/*z* 609.5 was the sodiated sodium carboxylates of the O-HS estolide, which was previously assigned by UHPLC-MS/MS. The MS/MS fragmentation spectrum of the parent ion 609.5 was consistent with that of the O-HS estolide obtained by UHPLC-MS/MS (not shown). The low-intensity signal at *m*/*z* 865.7 corresponded to the trimeric estolide P-HS-HS. The difference observed between ESI- and MALDI-based analyses should be ascribed to the diverse ionization efficiency of the species under conditions of the two ion sources. In agreement with UHPLC-MS/MS, MALDI-MS confirmed the absence of acylglycerols at detectable levels. The very low abundance of unmodified unsaturated FAs, intrinsically more oxidizable, confirmed that oil had undergone deep autoxidation.

### ^1^H- and ^13^C-NMR spectroscopy

**Fig. 3:**
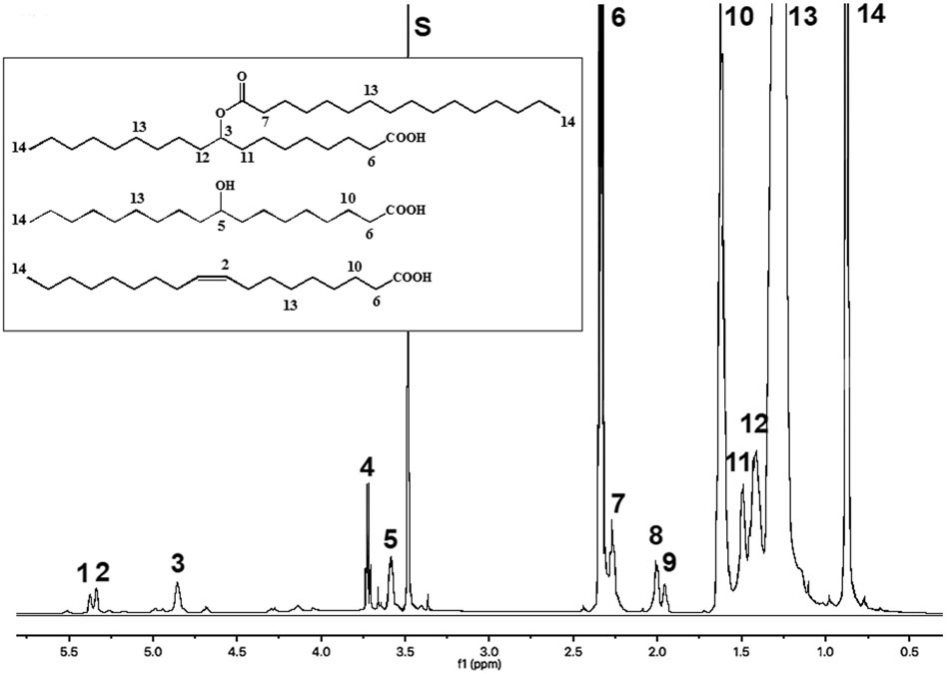
High-resolution 600 MHz ^1^H-NMR spectrum of lipid matter (Mann-S1). Numbers associate the resonances with the proton positions within representative structures displayed in the inset. Assignments are further detailed in Supplementary Material, Table S1. S: methanol residue.

**Fig. 4:**
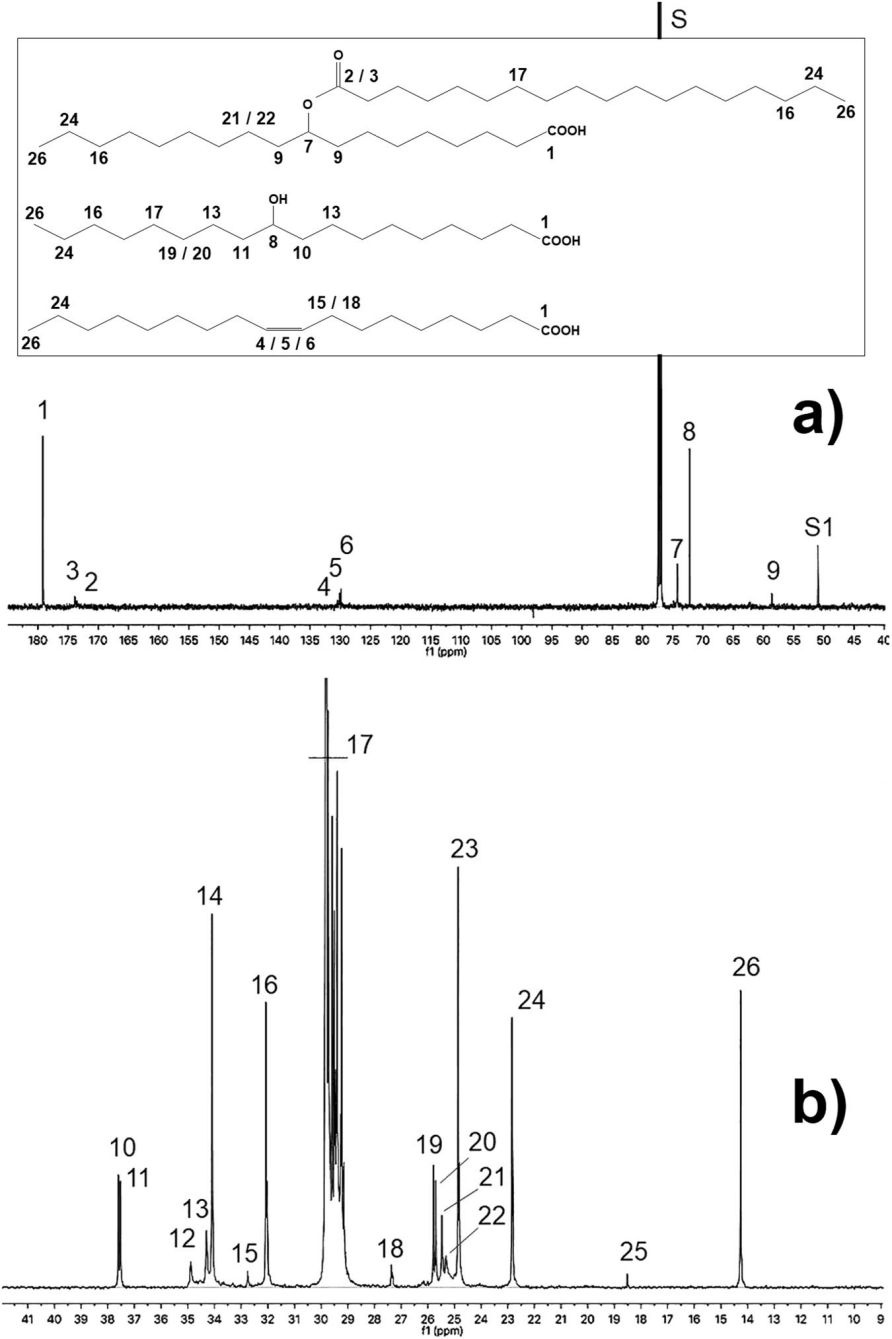
High-resolution 150.91 MHz ^13^C-NMR spectrum of lipid matter (Mann-S1). **a** Low and **b** high field regions at 180–50 ppm and 10–40 ppm, respectively. Numbers associate the resonances with carbons within representative structures displayed in the inset. Assignments are further detailed in Supplementary Material, Table S2. S: chloroform; S1: methanol residue.

The ^1^H- and ^13^C-NMR spectra **(**Figs. 3 and 4) clearly indicate the lipid nature of Mann-S1 because of the typical signals of acyl chains. The assignments are detailed in Supplementary Tables S1 and S2. Compared to the spectra of extra-virgin olive oil (EVOO) and the literature data^17^, the Mann-S1 lacked the characteristic complex proton multiplet at 5.18 ppm of the CH-2’ and the two quartets centered at 4.25 ppm and 4.10 ppm of the CH_2_-1’/CH_2_-3’ protons of the glycerol backbone. Accordingly, the ^13^C-NMR spectrum lacked the C1 ester carbonyl resonances at 173.3 ppm (*sn*-1,3) and 172.8 ppm (*sn*-2), as well as the glycerol carbons at 62.0 and 68.8 ppm for *sn*-1,3 and *sn*-2, respectively^17^. The presence of an intense carbonyl signal at 179.6 ppm corresponding to that of FAs and free acid estolides^18^ confirmed the virtually complete disappearance of acylglycerols and the conversion of a consistent part of them into free and hydroxy FAs, which in part condensed into estolides. The less intense resonances at 173–174 ppm matched those of the ester carbonyl carbons of estolides, which for oleic estolides are expected at 173.7 ppm^12^. The ^1^H resonance at 3.65 ppm should be assigned to the -OH proton of hydroxyl FAs rather than to the secondary alcohol of 1,3-diacylglycerols. Indeed, the primary alcohol proton of 1,2-di- and mono-acylglycerols at 3.99 ppm was missing. Intense ^13^C resonances of hydroxylated carbons of hydroxyacids and -CH-O-C=O- of estolides were found at 74 and 72 ppm^19^. The corresponding resonances of the ester methine detected as a broad signal at 4.83–4.86 ppm in the proton spectrum are also diagnostic of the estolide linkage^12^. The very low-intensity signals at 5.30–5.40 ppm and around 131 ppm in the ^1^H- and ^13^C-spectra, respectively, originating from the unsaturated carbon bonds, indicated that only a small percentage of unsaturated FAs had survived, due to the reduction or hydration of olefinic groups. We also detected diagnostic signals for *cis*- and *trans*-allylic carbons of monoenes (27.4 and 32.7 ppm, respectively), whose relative intensity (with respect to saturated FAs) was consistent with the GC data (2–3% of total FAs). Notably, no signals from free glycerol were detected, probably because it had evolved into volatile compounds (aldehydes, ketones, carboxylic acids) or reactive species, such as acrolein. In addition, glycerol is scarcely soluble in chloroform that has been used for NMR analysis.

The free acidity of Mann-S1 determined by microtitration was 68.87% (palmitic acid equivalents), which is an extremely high value for an oil, confirming that most triacylglycerols were hydrolyzed into free FAs. By contrast, peroxides were not detected; for a long-stored fat, this might be indicative of a sample at the final stage of its oxidative process.

### GC-flame ionization detector (FID) analysis of FAs

**Fig. 5:**
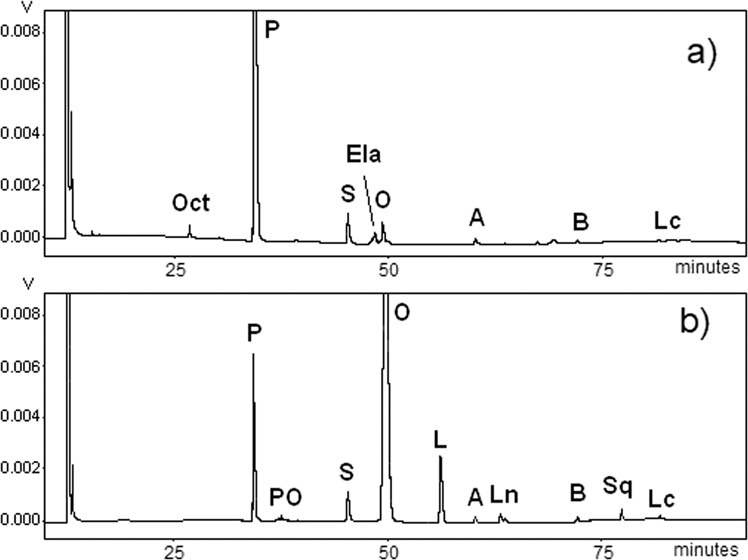
High-resolution gas chromatographic (HR-GC) profiles of fatty acid methyl esters (FAMEs) from sample Mann-S1. The GC chromatogram of Mann-S1 **a** is compared to that of a reference fresh olive oil **b**. Peak assignment: Oct octanoic acid (C8:0), P palmitic acid (C16:0), PO palmitoleic acid (C16:1n-7), S stearic acid (C18:0), Ela elaidic acid (C18:1n-9 t), O oleic acid (C18:1n-9 c), L linoleic acid (C18:2n-6 c,c), Ln linoleic acid (C18:2n-3 c,c,c), A arachidic acid (C20:0), B behenic acid (C22:0), Sq squalene, Lc lignoceric acid (C24:0).

The HR-GC profile of FAMEs was dominated by palmitic (C16:0) and stearic (C18:0) acids (Fig. 5a) accounting for 91% and 3.6%, respectively. Compared to the FA profile of a fresh EVOO sample (Fig. 5b), Mann-S1 contained very low amounts of oleic acid (C18:1*n*-9c) and its *trans*-isomer elaidic acid (C18:1*n*-9t) (2.3% and 1.5%, respectively), whereas linoleic acid (C18:2) and linolenic acid (C18:3) were not detected, because they oxidize at a rate almost 40- and 80-fold higher than oleic acid, respectively^20^. Evidently, the time-course evolution of FAs reflected the higher susceptibility of unsaturated FAs to autoxidation compared to saturated ones over about 20 centuries. The C16:0/C18:0 ratio of FAs was compatible to that of olive oil. Trace amounts of C20:0, C22:0, and C24:0 FAs also mirrored the relative ratios ordinarily found in olive oil (Fig. 5b). The hydroxy-FAs, expected from the LC-MS analysis, were not detected by GC on our polar capillary column^21^. Elaidic acid (C18:1,*n*-9*t*), not detected in fresh olive oil, is formed by heat-induced isomerization of the native *cis*-monoene (oleic acid). Very prolonged storage of vegetable oils at room temperature should not cause *cis*/*trans* isomerization, which is, instead, observed in heated, fried, and refined (at 180–250 °C) oils^22^. Thus the occurrence of elaidic acid at a level comparable to that of residual oleic acid could be the result of the thermal shock that the oil underwent during the eruption. Interestingly, the local temperature in the area invested by the eruptive ash has been reported to range between 240 and 370 °C, as calculated by optical analyses on charred wooden beams from Herculaneum (Villa dei Papiri)^23^. Other data inferred from the bone coloration of the skeletons of victims or from a recently described instantaneous vitrification of a human brain^24^ suggest that the local temperature might have exceeded 500 °C^25^.

### Attenuated total reflectance–Fourier transform infrared spectroscopy

**Fig. 6:**
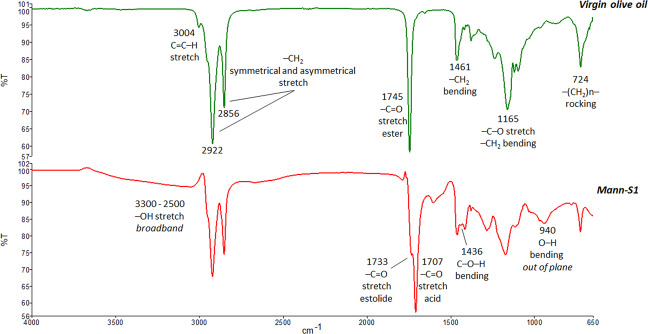
ATR-FTIR spectroscopic analysis. The spectrum of Mann-S1 (red line) was compared to that of a fresh virgin olive oil (green line). The strong carbonyl absorption of the triacylglycerol esters (1745 cm^−1^) detected in fresh oil was shifted to the typical carbonyl frequencies of free acids (1707 cm^−1^) and estolides (1733 cm^−1^).

ATR-FTIR spectroscopy is an effective and sensitive method for edible oil classification. It has been successfully exploited to monitor the molecular changes in terms of dominant functional groups occurring in severely oxidized oils^26^. The ATR-FTIR spectrum of Mann-S1 is compared to that of authentic EVOO in Fig. 6, which also reports the main absorption bands. Overall, the spectrum of Mann-S1 showed a strict similarity to that of EVOO, with both presenting a composition based substantially on long-chain FAs. By contrast, Mann-S1 showed the additional presence of -OH groups of both carboxylic acids and hydroxy FAs^6^. Compared to the EVOO, the carbonyl absorption of the triacylglycerol esters (1745 cm^−1^) was shifted to the frequency of free carboxylic acids (1707 cm^−1^). The shoulder at 1733 cm^−1^ is diagnostic of the estolide carbonyl^27^, thereby supporting MS and NMR identifications. The disappearance of the characteristic olefinic C=C stretch absorption (3004 cm^−1^) in Mann-S1 proved the almost complete saturation of the double bonds in the aliphatic chains of FAs. *Trans* double bonds exhibit diagnostic bands in the FTIR spectra of oils centered at 967 cm^−1 ^^22^. In this case, it was not possible to evidence the distribution of double bonds between the geometrical isomers, due to a low relative abundance of olefinic groups. The formation of free hydroxyl groups resulting from the hydration of double bonds and the release of free FAs was supported by the diagnostic absorption bands of C-O-H and O-H bending in the fingerprinting spectral region (Fig. 6b). Overall, the ATR-FTIR spectrum of Mann-S1 was compatible with the complete hydrolysis of acylglycerols and with the formation of free (hydroxy-) FAs and estolides, originating from their coupling.

### Analysis of sterols

Sterols represent a major class of compounds within the unsaponifiable fraction of fats and oils. Despite natural slight quantitative fluctuations, the sterol patterns distinctively fingerprint fats and oils, so that they are commonly used for detecting adulteration or assessing oil and fat authenticity^28^.

In the case in point, we sought to exclude the fact that Mann-S1 was fat of animal origin, such as lard. The GC-FID chromatogram of sterols is shown in Supplementary Material, Fig. S3. Sterols presented the typical pattern of the olive oil with β-sitosterol largely dominant over stigmasterol, Δ5-avenasterol, and campesterol, with the latter never exceeding 4% of the total sterol fraction in EVOO^28^. The prevalence of β-sitosterol combined with the absence of cholesterol at a significant amount confirmed the plant origin of the fat. Interestingly, the very intense peaks eluting at high retention times were most likely oxidized phytosterols, such as 7-hydroxysitosterol and 7-ketositosterol.

### Analysis of volatile organic compounds (VOCs) by SPME-GC/MS

**Fig. 7:**
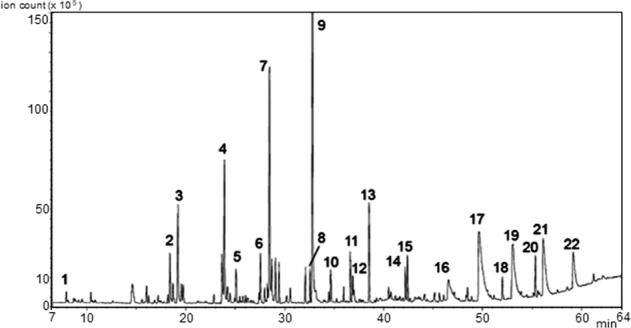
SPME GC/MS chromatogram (TIC) of VOCs in Mann-S1. Peak identification: (1) octane, (2) decane-3,7-dimethyl, (3) hexanal, (4) heptanal, (5 and 6) hexadecane, (7) octanal, (8) nonanone, (9) nonanal, (10) heptanol, (11) 2-decanone, (12) decanale, (13) 1-octanol, (14) 1-nonanol, (15) 2-(E)-decenal, (16) pentanoic acid, (17) hexanoic acid, (18) gamma-butyl-butyrolactone, (19) heptanoic acid, (20) gamma-decalactone, (21) octanoic acid, and (22) nonanoic acid.

The complex pattern of VOCs profiled by SPME-GC/MS (Fig. 7) was dominated by the C_7_–C_9_ aliphatic aldehydes (nonanal, octanal), hydrocarbons, aliphatic alcohols (e.g., 1-octanol) and C_5_–C_9_ aliphatic acids (pentanoic, hexanoic, heptanoic, octanoic, and nonanoic acid), therefore reflecting the panel of markers for the rancidity of olive oil^20,29^. The complex assortment of VOCs is the result of a severe autoxidation that has interested especially unsaturated FAs (i.e., oleic and linoleic acids), thus allowing for confirmation that the original FA composition was dominated by oleic acid as in olive oil. In general, the identification of plant oils in archeological finds is challenging because FAs suffer from a high oxidation rate and, often, the breakdown products cannot be straightforwardly assigned to the parent FAs^30^. However, while predominant volatile C_5_–C_6_ compounds or unsaturated long-chain aldehydes are indicative of linoleic (ω6)-rich oils, high relative amounts of C_7_, C_8_, and C_9_ volatile compounds, as in this case, are preferential products of the oxidative decomposition pathway of oleic-acid-rich oils^20^. In particular, nonanal, the most abundant volatile compound here detected, is a specific degradation compound arising from oleic acid hydroperoxides. Consistent with other findings, the profile of VOCs trapped in the organic matter supports the fact that Mann-S1 was in origin olive oil that has undergone drastic oxidation. The whitish-yellow color of the organic residue (Fig. 1e) proves that parallel oxidation has involved the natural olive oil pigments (e.g., carotenes and chlorophylls) as well.

### Radiocarbon dating

Sample code, laboratory code, carbon contents, carbon stable isotope ratio, ^14^C concentration (percentage of Modern Carbon), radiocarbon age (Before Present), and calibrated age (1*σ* and 2*σ* A.D., unless otherwise specified) are reported in Table S3 (Supplementary Material). Stable isotope measurement confirms the nature of the sample as a C3 plant. The calendarial estimated age of the organic material is between 51 B.C. and 176 A.D. (Fig. S4 in Supplementary Material). Therefore, radiocarbon dating places Mann-S1 within the right timeframe corresponding to the eruption of 79 A.D. and authenticated the specimen as an original archeological olive oil of the Roman Imperial age.

## Discussion

Similar to biological entities, foods are complex (bio)chemical systems encrypting information distinctive of the raw material identity, its processing, and its storage status. Modern analytical techniques are currently able to decipher the molecular code concealed in archeological food matter, contributing to the development of “archeometry,” which has anthropological and scientific relevance. Archeological lipid residues, often found in small quantities, may have changed significantly over time because natural oxidation processes alter organic matter, forming a heterogeneous panel of compounds covering a large range of molecular weight, from volatile components up to high-molecular-weight polymers. Modern analytical techniques enable the effective characterization of the degradation products of archeological organic materials in order to also infer their native composition.

Compared with previous works, which only offered clues to establish the vegetable nature of the fats retrieved in the archeological sites of Pompeii and Herculaneum, our results strikingly highlight the molecular evolution of olive oil through an almost 2000-year-long storage period. Mann-S1 is probably the most ancient olive oil residue recovered (and currently exposed) in a significant amount. The containing bottle is amazingly similar to the one depicted in a Roman fresco also hosted at the MANN (Fig. S1).

Chemical analyses disclosed the signature of modifications that occurred in olive oil due to both *una tantum* exposure to extreme heat (formation of *trans* FAs) and a slow, deep biomolecular evolution over almost 20 centuries. Based on convergent data obtained from multiple analytical techniques, Mann-S1 was authenticated as a deeply altered olive oil of the Roman Imperial age, in which acylglycerols have been completely hydrolyzed into free FAs. Unsaturated bonds have been almost completely saturated by conversion into hydroxy FAs, which, in turn, have partly esterified other free FAs to form estolides.

In research carried out over 20 years ago, we detected signs of a similarly lipolyzed and oxidized vegetable ointment in a small oil sample contained in a little glass ampoule from Pompeii^4,31^.

Analyzing a sample similar to Mann-S1, Ribechini et al.^6^ realized that the chemical nature of the oil was deeply modified. However, their hypothesis that oil was pretreated with lead oxide (*litargirium*) to yield soaps appears to be incompatible with the presence of solidified fat in the narrow-neck bottle. According to our findings, the relative enrichment of saturated FAs and the formation of estolides have contributed to solidify the olive oil in situ, which assumed the current shape most likely because the bottle lay inclined during its dormancy (Fig. 1b).

The assessment of olive oil in bottles of ordinary daily use 2000 years ago (Fig. S1), similar to that used nowadays, further emphasizes the dietary importance of olive oil in the Mediterranean Basin and in “Campania Felix” since ancient times.

The definition of chemical details reinforces the relevancy of the remains hosted at MANN and provides new clues for characterizing oil-containing archeological samples.

## Methods

### Archeological sample

Archeological report no. 84847 (Mann-S1) from the MANN (National Archaeological Museum of Naples, Naples, Italy) collection is a 25.5-cm-high cylindrical glass bottle with a single handle and a short neck (Inv. 848747; ASSAN, reg. 98; AdSN, 396/2.; Vitrum)^2^. The bottle, likely coming from Herculaneum and hosted in the MANN collection, was half-filled with yellowish waxy organic matter, which solidified in a clarinet-spout shape (Fig. 1a). Interestingly a fresco coming from Praedia of Giulia Felice in Pompeii depicts a very similar bottle containing a liquid (Fig. S1), suggesting, for the bottle, a temporal placing at the Roman Imperial age. Mann-S1 was sampled from the inner part using a sterile stainless-steel spatula, after scraping of the upper brown surface (Fig. 1c), aliquoted, and stored at −20 °C in sealed glass vials under N_2_ until analyses.

Part of the sample (2 g), dissolved in 5 mL choloroform:methanol (2:1, v–v), was filtered on disposable nylon 0.22-μm filters (Merck-Millipore, Burlington, MA, USA), aliquoted in glass vials, and dried under nitrogen flux at room temperature (Fig. 1d).

### Free acidity and peroxide index

Free acidity was determined by microtitration, adapting the conventional method. Briefly, 10 mg of sample dissolved in diethyl ether:ethanol was titrated with N/100 NaOH using phenolphthalein as the indicator. Values are expressed as palmitic acid equivalents, with C16:0 being the most abundant FA in the analyzed sample. The iodometric determination of total peroxide was performed using a 100-mg sample, with all reagents at 10% of the concentration recommended for the standard procedure^32^.

### Ultrahigh performance liquid chromatography–tandem mass spectrometry

The lipid sample (10 mg) was dissolved in 1 mL of CHCl_3_/CH_3_OH (2:1) at room temperature and washed twice with deionized water. The aqueous layer was discarded after checking for the absence of lipids by thin-layer chromatography. The organic layer was dried under N_2_ stream, reconstituted in CH_3_OH (100 µg mL^−1^), and directly analyzed by UHPLC-MS/MS according to Cutignano et al.^33^. Briefly, chromatographic separations were achieved on an Infinity 1290 UHPLC System (Agilent Technologies, Santa Clara, CA, USA), equipped with a Kinetex Biphenyl 2.6 μm, 150 × 2.1 mm column (Phenomenex, Castel Maggiore, Bologna, Italy) at 28 °C. Eluents A and B were LC-MS-grade water and methanol, respectively. The elution program consisted of a gradient from 40 to 80% B in 2 min, then to 100% B in 13 min, holding at 100% B for 7 min. A post-run equilibration step of 5 min was included before each analysis. The flow rate was 0.3 mL min^−1^. The injection volume was 10 μL and the autosampler was maintained at 10 °C. MS analysis was performed on a Q Exactive Hybrid Quadrupole-Orbitrap mass spectrometer (Thermo Scientific, San Jose, CA, USA) equipped with a HESI source. Source parameters were as follows: spray voltage positive polarity 3.2 kV, negative polarity 3.0 kV, capillary temperature 320 °C, S-lens RF level 55, auxiliary gas temperature 350 °C, sheath gas flow rate 60, auxiliary gas flow rate 35. Full MS scans were acquired in the range of 200–1200 *m*/*z* at 70,000 of mass resolution. For MS/MS analysis, a data-dependent ddMS2 Top 10 method was used. Mass fragmentation was obtained with a stepped normalized energy of 25–28–35% and 20–40% in positive and negative ionization mode, respectively.

### Matrix-assisted laser desorption ionization–time of flight MS

Before MS analysis, 5 mg of the lipid extract was dissolved in 1 mL of CHCl_3_ and vigorously vortexed with 1 mL of 0.5 M aqueous sodium acetate to promote the ionization of acylglycerols as Na^+^ adducts. After the separation of the biphasic system, the organic layer was used for the analysis. MALDI-TOF spectra were acquired on an UltraflexExtreme mass spectrometer (Bruker Daltonics) equipped with the FlexControl software package (version 3.4, Bruker Daltonics). Experiments were performed in the reflector positive ion mode using 10 mg mL^−1^ 2,5-dihydroxybenzoic acid in 50% aqueous acetonitrile (v/v) containing 5 mM sodium acetate as the matrix. External mass calibration was performed with a separate acquisition of standard TAG (obtained from Sigma-Aldrich). Analyses were carried out in triplicate to check for repeatability.

### NMR spectroscopy

A 10 mg aliquot of the sample was dissolved in 1 mL of chloroform-d (Fluka, Bucks, Switzerland) for NMR analysis. One-dimensional proton (^1^H) and carbon (^13^C) NMR spectra were recorded on a Bruker Avance III–600 MHz spectrometer (Bruker BioSpin GmbH, Rheinstetten, Germany), equipped with a TCI CryoProbe^TM^ fitted with a gradient along the *Z*-axis, at a probe temperature of 27 °C. ^1^H NMR spectra were acquired at a proton frequency of 600 MHz adding 516 transients of 16,384 points with a spectral width of 14 ppm. Time-domain data were all zero-filled to 32,768 points, and before Fourier transformation, an exponential multiplication of 0.8 Hz was applied. ^13^C NMR spectra were acquired at a frequency of 150.90 MHz adding 16,384 transients of 32,768 points, and before Fourier transformation, an exponential multiplication of 2.0 Hz was applied. Resonances of lipids were compared to those occurring in NMR spectra of fresh EVOO (Azienda Agricola Le Tore, Massa Lubrense, Naples, Italy) and assigned combining data from one-dimensional DEPT-^13^C NMR experiments, two-dimensional NMR (total correlation spectroscopy, correlation spectroscopy, heteronuclear single quantum correlation), and literature data^5,14,17,31,34^.

### HR-GC of FAs

FAMEs were obtained from 0.5 mg of an anhydrous sample with 1 mL of methanol/HCl 1 N 93/7 overnight at 50 °C in a sealed vial^35^. Methanol was evaporated under nitrogen flux and FAMEs were dissolved in 1 mL *n*-hexane for GC analysis, which was carried out using a Shimadzu GC-17A gas chromatograph (Shimadzu Italy, Milan) equipped with a FID and a SUPELCO capillary column SPTM-2560 (75 m length, 0.14 μm i.d. with 0.18 μm coated with poly (bis-cyanopropyl) siloxane (Supelco, New Haven, CT, USA). The oven temperature was held at 200 °C for 5 min, increased at a rate of 2 °C min^−1^ until 230 °C, and finally held for 30 min. Injector temperature and FID temperature: 240 °C. Carrier gas: Helium. Column flow: 0.3 mL min^−1^. Split ratio: 1/100. Injected volume: 2 μL. Peaks were assigned matching the retention times of FAMEs with those of pure standard compounds (mixture of pure FAMEs, Larodan, Malmoe, Sweden) injected under the same conditions. The acquisition software was the Class-VP data system version 4.6. (Shimadzu Italy, Milan).

### Attenuated total reflectance–Fourier transform infrared spectroscopy

ATR-FTIR analyses were performed using a Spectrum 400 spectrophotometer (PerkinElmer, Waltham, MA, USA), equipped with a deuterated triglycine sulfate detector. Mann-S1 was dissolved at a concentration of 5 mg mL^−1^ in CHCl_3_/methanol 2:1 (v/v). A drop of the resulting solutions was spotted onto the ATR diamond crystal and the solvent was air-dried. Overall, 32 scans/spectrum were acquired in the 4000–650 cm^−1^ range with a resolution of 4 cm^−1^. EVOO was used as a reference. Spectra were obtained in triplicate, averaged, and elaborated using the PE Spectrum software version 10.5.1, purchased with the instrument.

### Analysis of the sterol fraction

Aliquots (10 mg) of Mann-S1 were saponified with 10 mL of 2 M KOH/CH_3_OH heated to reflux for 30 min. The unsaponifiable components were extracted in diethyl ether and washed with deionized water until neutral reaction. The ether phase was dehydrated using anhydrous sodium sulfate, filtered on paper, and dried under a vacuum. Residual oil was dissolved in 0.2 mL of *n*-hexane and underivatized sterols were separated using an Agilent Technologies 6850 Series II gas chromatographer (Agilent Technologies, Palo Alto, CA, USA) equipped with a split/splitless injector and an FID, mounting an RTX-5, 30 m × 0.25 mm × 0.25 µm column (Restek, Bellefonte, PA, USA). Sample (1 μL) was transferred into screw-top autosampler vials for subsequent injection in split mode with a 1:5 ratio at 250 °C. The oven temperature program started at 200 °C (held for 2 min) and linearly increased at 20 °C min^−1^ to a final temperature of 300 °C. Plant sterol mix (Matreya, State College, PA, USA) was used as the external standard for qualitative and quantitative determinations. Data were recorded and processed using the ChemStation software (Agilent Technologies).

### Dynamic headspace–SPME and GC/MS of volatile compounds

The analysis of volatile compounds was carried out by SPME-GC/MS according to Sacchi et al.^36^. Two grams of sample were added to a 10-mL vial and heated at 40 °C. After 10 min of the equilibration phase, the fiber was exposed for 30 min. The SPME device (Supelco Co., Bellefonte, PA, USA) was equipped with a 50/30-μm thickness divinylbenzene/carboxen/polydimethylsiloxane fiber coated with a 2-cm-long stationary phase. Volatiles were analyzed by GC coupled with an MS using a GC/MS Shimadzu Model QP5050A instrument (Kyoto, Japan) equipped with a Supelcowax-10 capillary column (60 m, 0.32 mm i.d., with 0.5 μm thickness; Supelco Co., Bellefonte, PA, USA). The temperature was set at 40 °C for 4 min, followed by an increase of 3.5 °C min^−1^ up to 240 °C. That temperature was held for 3 min. The injector was kept at 230 °C. Helium was used as the carrier gas (1.4 mL min^−1^). Thermal desorption of VOCs was carried out by exposing the SPME fiber in the injector for 10 min. The identification of volatiles was performed by comparing retention times and mass spectra obtained by analyzing pure reference compounds under the same conditions. Moreover, the identification was confirmed by comparing mass spectra with those of the NIST database. Mass spectra were recorded at 70 eV. The source temperature was 200 °C and the interface temperature was 250 °C. Before use, the fiber was conditioned at 270 °C for 1 h for the analysis. Before each analysis, a blank test was performed to prevent the release of undesirable compounds.

### Radiocarbon dating

An 8 mg aliquot of Mann-S1 was pretreated according to the classical acid–alkali–acid protocol^37^ at the iCONa Lab of the University of Campania, where stable isotope measurement was also carried out. Accelerator MS radiocarbon dating was performed at the INFN-LABEC CHNet (Florence, Italy)^38^. Radiocarbon conventional dates were calibrated by using OxCal 4.3^39^ and the recommended calibration curve IntCal13^40^.

## Supplementary information

Supplementary Information

## Data Availability

The authors declare that data supporting the findings of this study are available within the paper and its supplementary information files. Raw data are available from the corresponding author upon reasonable request.
