# Supplementary material for: Olive oil from the 79 A.D. Vesuvius eruption stored at the Naples National Archaeological Museum (Italy)
Source: NPJ Sci Food. 2020 Nov 2;4:19. doi: 10.1038/s41538-020-00077-w (PMC7606599; doi:10.1038/s41538-020-00077-w)
Supplement: Supplementary file 1 — Supplementary Information [file 41538_2020_77_MOESM1_ESM.pdf]

Supplementary Materials

Olive oil from the 79 A.D. Vesuvius eruption stored at the Naples National Archaeological Museum (Italy)

Raffaele Sacchi*, Adele Cutignano, Gianluca Picariello, Antonello Paduano, Alessandro Genovese, Francesco Siano, Genoveffa Nuzzo, Simonetta Caira, Carmine Lubritto, Paola Ricci, Alessia D'Auria, Gaetano Di Pasquale, Andrea Motta, Francesco Addeo

*Correspondence to: sacchi@unina.it

This PDF file includes:

Supplementary Text
Figs. S1 to S4
Tables S1 to S3

Supplementary Text: description by Prof. Covelli (1827)

[…. that kind of butyric mud in which the olives lie is brownish-yellow, soft like butter, has a strong rancid odor, stains paper similarly to fixed oils and fatty substances. The butyric mud will melt upon gentle heating (60° to 70°C) and if heated on a platinum leaf, it burns with a beautiful white flame with no residue other than that of an ash in the shape of a very light white petiole flake for which a faint breath will take away. With alkalis it forms soaps; distilled in vessels closed by hydrogen carbonate gas, acetic acid, carbonic acid, carbonic oxide gas and a carbonaceous residue. This butyric substance, tested with the Chevreul method, was found to be comprised of large amounts of oleic acid, a small amount of margaric acid, and a substance similar to the sweet principle of fixed oils, but which also differs in many characteristics, and so, could be a new product, and finally, the butyric substance is comprised of a very small quantity of earthy substance that comes from the volcanic ash that fills the upper part of the pot "..." Examination of the butyric substance in the narrow-mouthed pot: this substance is much softer than the antecedent. It has a yellowish-green color, a strong rancid odor, and in its mass is comprised of many small globules that are similar to fish eggs, but which the strong lens cannot well determine. In summary, this substance is entirely analogous to the substance found within olives. It is comprised of the same principles, although it contains a greater proportion of oleic acid, and of the indeterminate substance that is similar (in some ways) to the sweet principle of fixed oils. It seems that it was originally nothing but olive oil, containing some kind of vegetable rather than animal sauce, since its distillation did not provide nitrogen compounds] (Covelli, 1827).

"*… quella specie di fango butiroso nel quale giacciono le olive è giallo-bruniccio, molle come il butiro, ha odor forte di rancido, macchia la carta come gli olj fissi e le sostanze grasse; fondesi ad un dolce calore (60° a 70° centig.), riscaldato su la foglia di platino, brucia con bella fiamma bianca senz'altro residuo che quello di un cenere della forma di picciol fiocco bianco leggerissimo che un debole soffio porta via; con gli alcali forma de' saponi; distillato in vasi chiusi da gas idrogeno carbonato, acido acetico, acido carbonico, gas ossido di carbonico ed un residuo carbonoso. Questa sostanza butirosa, saggiata col metodo di Chevreul si è trovata composta di acido oleico in gran quantità, di picciola dose di acido margarico, e di una sostanza analoga al principio dolce degli olj fissi, ma che ne differisce per molti caratteri, e che potrebb'essere un nuovo prodotto, e finalmente di sostanza terrosa in piccolissima quantità, proveniente dal cenere vulcanico che riempiva la parte superiore del vaso"…* "*Esame della sostanza butirosa del vaso a bocca stretta: questa sostanza è molto più molle dell'antecedente; essa ha un color verde-giallognolo; ha forte odor rancido, e mostra nella massa tanti globicini bigi, simili ad uova di pesce, ma che la lente forte non può ben determinare. Questa sostanza in somma è interamente analoga a quella trovata colle olive; essa è composta degli stessi principi, quantunque contenesse maggiore quantità di acido oleico, e di quella sostanza indeterminata analoga in qualche modo al principio dolce degli oli fissi. Sembra che essa in origine non sia stato altro che olio di olive contenente qualche salsa piuttosto vegetabile che animale, poiché la sua distillazione non ha dato composti di azoto*" (Covelli, 1827).

Covelli N. Rapporto su le olive e su la sostanza butirosa trovate in Pompei il dì 4 agosto del 1826. In: *Musei Borbonico III* , Napoli (1927).

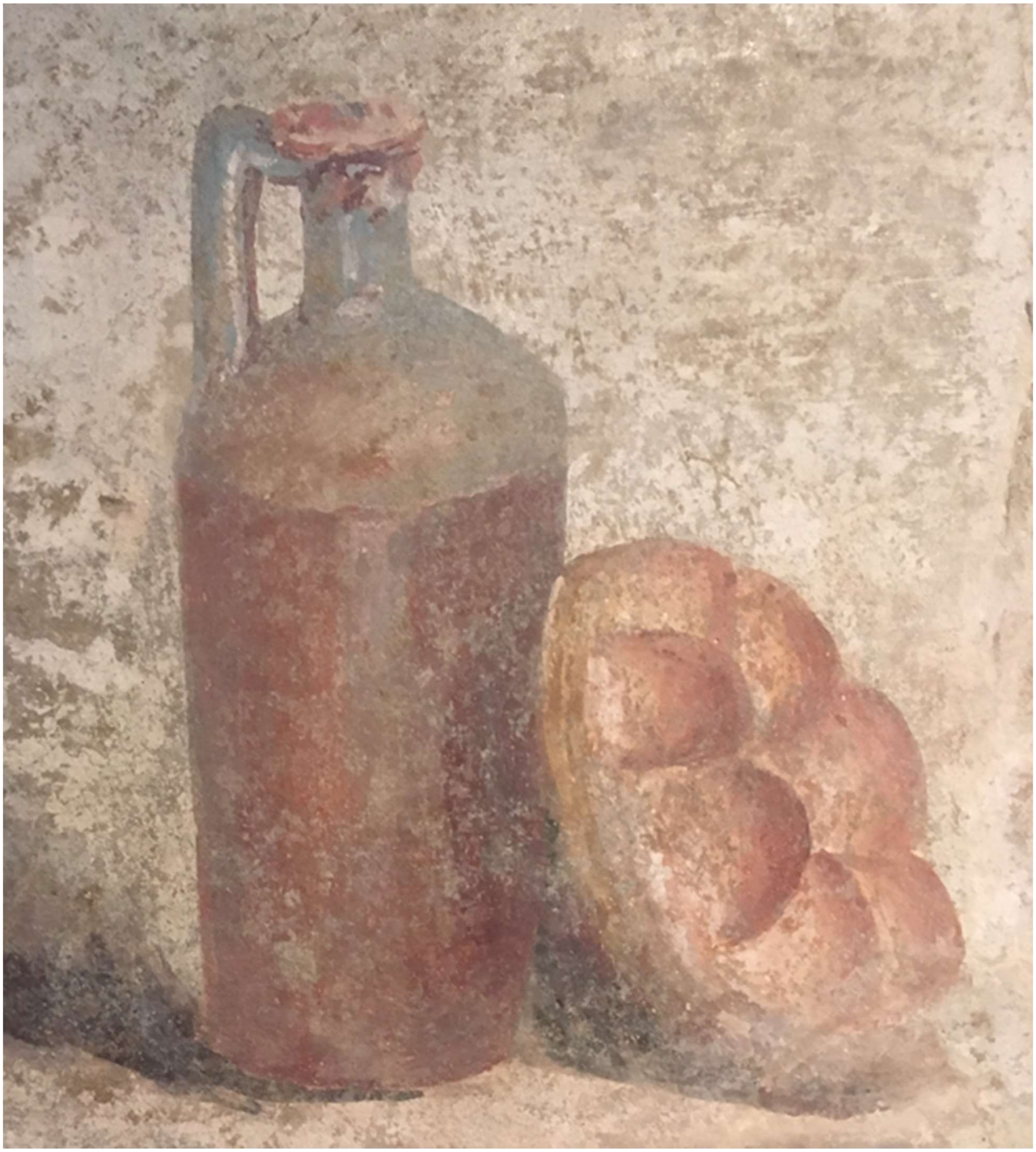

Fig. S1
Fresco coming from Praedia of Giulia Felice (Pompeii archaeological area) with a charred loaf and a bottle with the same shape of that containing the organic matter (solidified olive oil) studied in the present work.

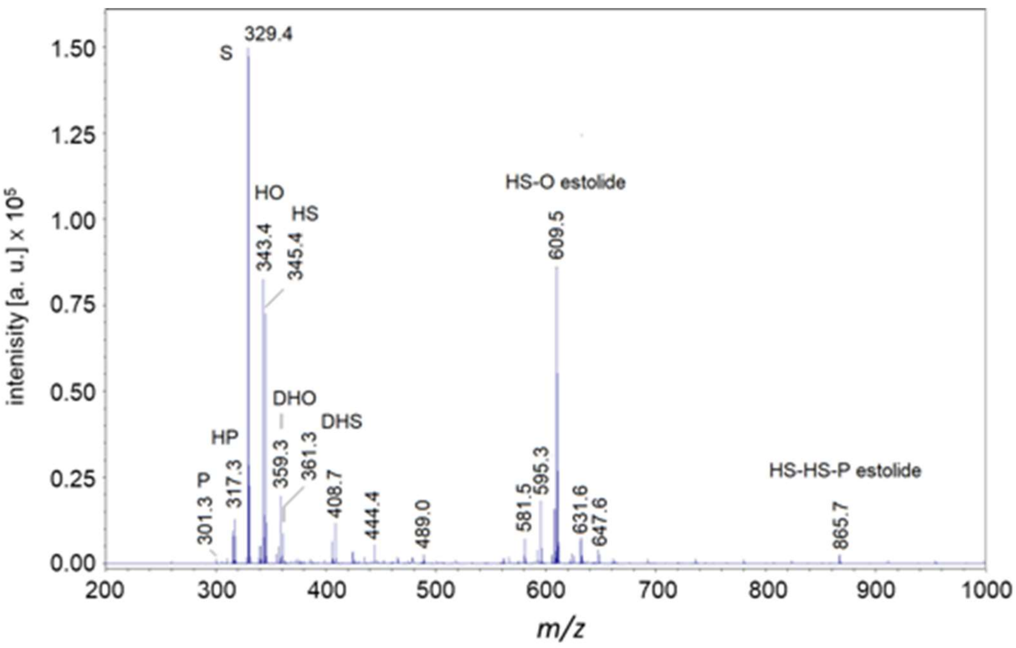

Fig. S2
MALDI-TOF mass spectrum of underivatized lipids from Mann-S1. The spectrum is dominated by signals of free (hydroxy) FAs and of O-HS estolide. A trimeric estolide was clearly detected and glycerol-based lipids were missing.

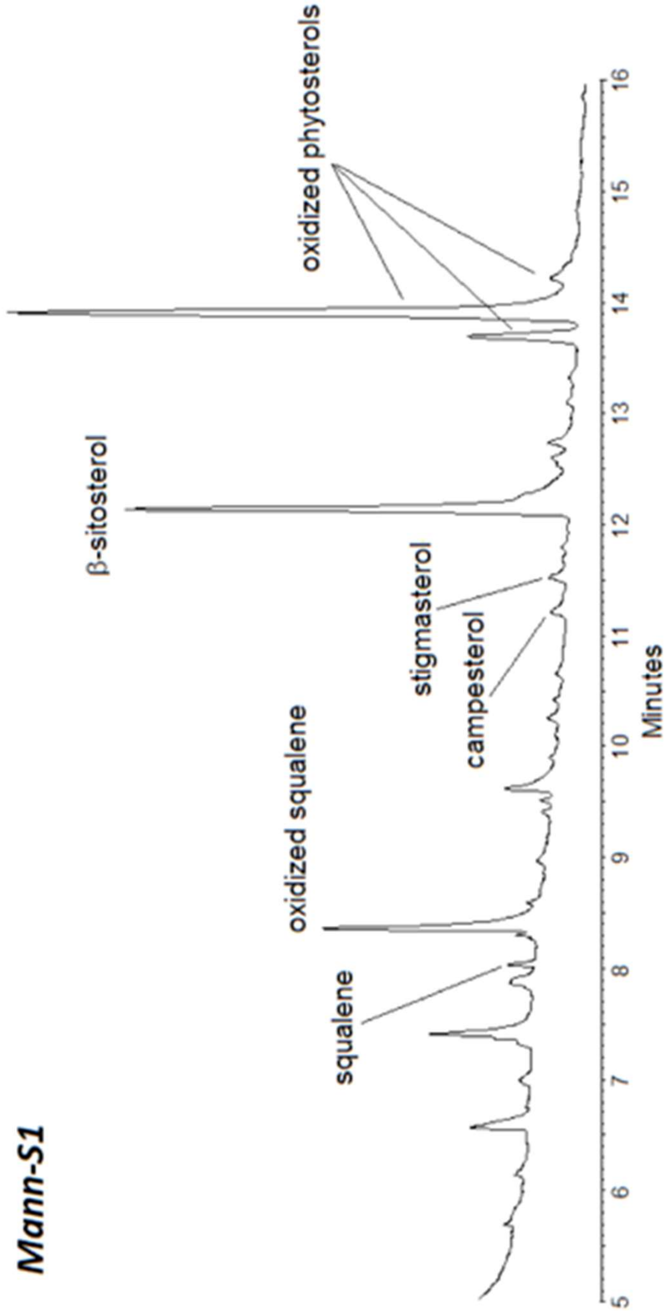

Fig. S3 GC-FID analysis of the unsaponifiable fraction of Mann-S1. β-sitosterol was largely dominant, which was expected for an olive oil.

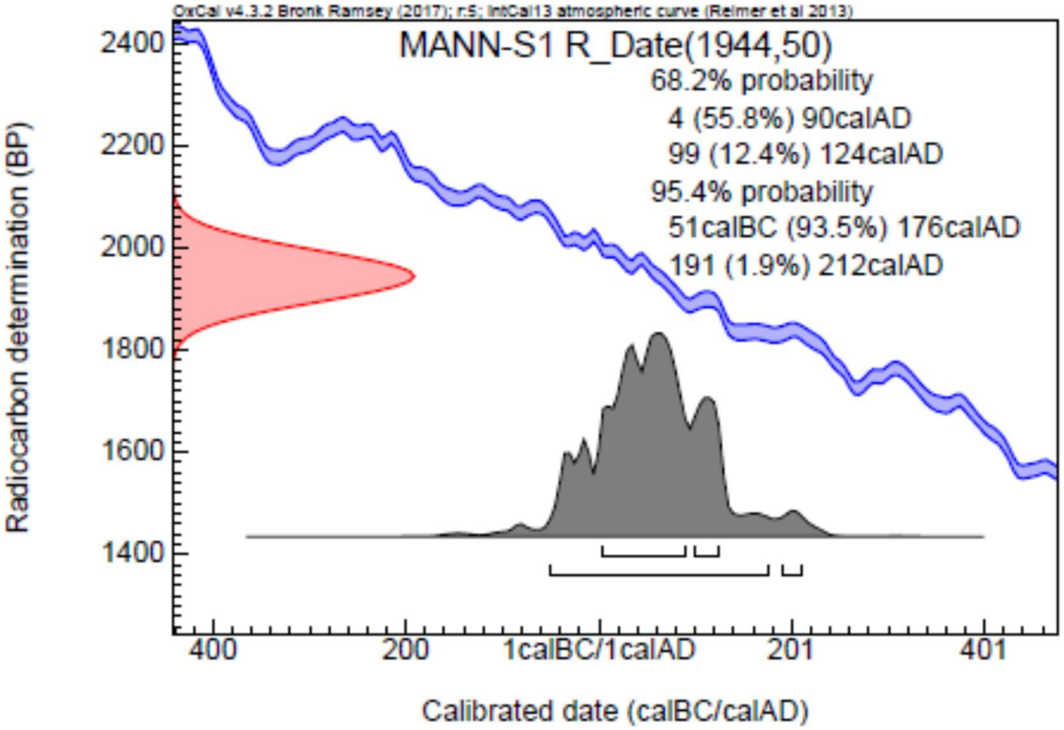

Fig. S4 Radiocarbon dating calibration.

Table S1 Chemical shift (δ, ppm/TMS) assignment of the ^{1}H-NMR spectrum (Fig. 3).

Signal number	δ (ppm/TMS)	Proton, Compound (Ref)
1	5.38	CH=CH, estolides (Cermak & Isbell, 2001)
2	5.33	-CH=CH-, unsaturated fatty acids
3	4.84	C-H ester methine estolides (Cermak & Isbell, 2001)
4	3.72 (quartet)	unassigned
5	3.59 (multiplet)	-CH-OH, hydroxy-acids
S	3.51 (singlet)	CH_3-OH, extraction solvent
6	2.32 (multiplet)	-CH_2 C-1 acyl chain, all free fatty acids
7	2.28 (triplet)	α-methylene, estolides (Cermak & Isbell, 2001)
8	2.05	unassigned
9	1.85	unassigned
10	1.65	-CH_2 (C2 acyl chain), all acyl chains
11	1.35	-CH_2 estolides (Cermak & Isbell, 2001)
12	1.44	-CH_2 estolides (Cermak & Isbell, 2001)
13	1.31 (broad m.)	internal -CH_2 (from C3 to ω-2), all acyl chains
14	0.87 (triplet)	-CH_3 (ω-1), all acyl chains

Table S2 Chemical shift (δ, ppm/TMS) assignment of the ^{13}C-NMR spectrum (Fig. 4).

Signal number	δ (ppm/TMS)	Carbon, compound
1	179.2	C-1, free fatty acids
2	173.9	C-1, estolide esters
3	173.5	C-1, estolide esters
4-6	129.9-130.4	C-9 and C-10, n-9 MUFA (*cis* and *trans*)
7	74.3	CH-methine, estolides
8	72.2	CH-methine, hydroxyacids
9	58.6	CH_2- estolides
S1	50.9	CH_3-OH extraction solvent
10	37.6	-CH_2 hydroxyacids
11	37.5	-CH_2 hydroxyacids
12	34.9	C-2 estolides
13	34.3	C-2 hydroxy-acids
14	34.1	C-2, free fatty acids
15	32.7	C-8 and C-11, *trans* n-9 MUFA acyl chains
16	32.1	-CH_2 ω-3, all acyl chains
17	29.0-29.8	C-4 to ω-4 internal -CH_2, all acyl chains
18	27.3	C-8 and C-11, *cis* n-9 MUFA acyl chains
19	25.8	-CH_2- Cγ hydroxyacids
20	25.7	-CH_2- Cγ hydroxyacids
21	25.5	-CH_2- C-2 estolides
22	25.3	-CH_2- C-2 estolides
23	24.9	-CH_2 C-3 all fatty acyl chains
24	22.8	-CH_2, ω-2 all fatty acids
25	18.5	-CH_2, unassigned
26	14.3	-CH_3, ω-1 all fatty acids

Table S3 Stable isotope ratios and radiocarbon determination on Sample Mann-S1 (BP).

**Sample code**	**Lab. Code**	**C Content C (%)**	**$\delta^{13}C$ (%)**	**Concentration ^{14}C(pMC)**	**RC age - $t_{rc(years\ BP)}$**	**Calendarial Age (years AD – 1σ)**	**Calendarial Age (years – 2σ)**
Mann-S1	Fi4274	98.4	-28,0 (0,1)	78.51 ± 0.53	1944± 50	**[4-90]** [99-124]	**[51BC- 176AD]** [191-212]
